# A Poroelastic Model of a Fibrous-Porous Tissue Engineering Scaffold

**DOI:** 10.1038/s41598-018-23214-8

**Published:** 2018-03-22

**Authors:** Daniel Yuan, Sarah M. Somers, Warren L. Grayson, Alexander A. Spector

**Affiliations:** 1 Department of Biomedical Engineering, Johns Hopkins University, Baltimore, MD USA; 2 Translational Tissue Engineering Center, Johns Hopkins University, Baltimore, MD USA; 3 Institute for NanoBioTechnology, Johns Hopkins University, Baltimore, MD USA; 4 Department of Materials Science and Engineering, Johns Hopkins University, Baltimore, MD USA

## Abstract

Tissue engineering scaffolds are used in conjunction with stem cells for the treatment of various diseases. A number of factors provided by the scaffolds affect the differentiation of stem cells. Mechanical cues that are part of the natural cellular microenvironment can both accelerate the differentiation toward particular cell lineages or induce differentiation to an alternative cell fate. Among such factors, there are externally applied strains and mechanical (stiffness and relaxation time) properties of the extracellular matrix. Here, the mechanics of a fibrous-porous scaffold is studied by applying a coordinated modeling and experimental approach. A force relaxation experiment is used, and a poroelastic model associates the relaxation process with the fluid diffusion through the fibrous matrix. The model parameters, including the stiffness moduli in the directions along and across the fibers as well as fluid diffusion time, are estimated by fitting the experimental data. The time course of the applied force is then predicted for different rates of loading and scaffold porosities. The proposed approach can help in a reduction of the technological and experimental efforts to produce 3-D scaffolds for regenerative medicine as well as in a higher accuracy of the estimation of the local factors sensed by stem cells.

## Introduction

Scaffolds, a key part of regenerative medicine, control the microenvironment for adhesion, migration, proliferation, and differentiation of cells inside^1^ (for review). Fibrous-porous scaffolds mimicking the natural structure of tissue are effectively used in applications like skeletal muscle and tendon. Such scaffolds often have a fibrin-based fibrous component mixed with another polymer which is then dissolved, providing a porous structure of the scaffold. Recently, electrospun fibrin-alginate composite scaffolds with tunable longitudinal stiffness and alginate volume fraction have been proposed^2–4^.

One critical function of the scaffolds is control of stem cell differentiation. Mechanical factors play an important role in this function since they can provide both the acceleration of stem cell differentiation toward a given lineage^5,6^ and regulation of lineage fate^7,8^. Mechanical stimulation can be exerted through externally applied stresses/forces and strains, innate physical properties of the extracellular matrix (scaffold), or combinations of both. The application of cyclic unidirectional strains improves myogenesis of several types of stem cells^9–12^. Stiffness of the extracellular matrix (ECM) directs stem cell differentiation toward neurogenesis, myogenesis, and osteogenesis, within ranges of 0.1–1 kPa, 8–17 kPa, are 25–40 kPa typical to brain, muscle, and bone tissues, respectively^13,14^. The viscoelastic properties of ECM, stress relaxation time^15,16^ and loss modulus^17,18^, affect stem cell differentiation (osteogenesis). The bulk and surface properties of the ECM also affect stem cell differentiation whereby the fibrous structure^19^ or grooved topography^20^ of the scaffolds promotes stem cell alignment and improve differentiation.

**Figure 1:**
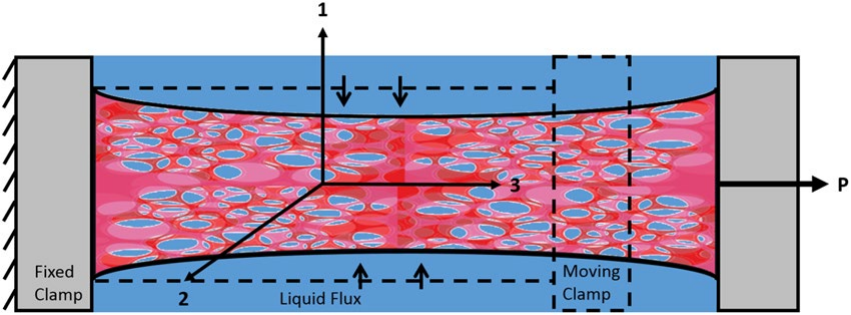
Sketch of the force relaxation experiment made with a cylindrical sample of the fibrous-porous scaffold. The elastic properties of the scaffold materials are the same in the *x*_*1*_*x*_2_-plane, but they are different from those in the fiber direction, *x*_*3*_ (transversely isotropic material). The scaffold material is a mixture of two phases, fluid and solid. Under the action of the force *P*, the cylinder extends resulting in the fluid going inside the cylinder. Such diffusion of the fluid explains the relaxation of the applied force.

In the present paper, we focus on the mechanical properties of fibrous-porous scaffolds. We propose a model (also ref.^21^) of such scaffolds and use the experimental data to estimate the model parameters. In the supporting experiment (Fig. 1), cylindrical specimens are strained with a fixed rate up to 10% after which the strain is kept fixed. The time course of the corresponding stress (load intensity) during two stages, loading and relaxation, is recorded and used for the estimation of the model parameters. The proposed model treats the scaffold as a long linear poroelastic transversely isotropic cylinder whose material parameters are two Young’s moduli (along the fiber direction and in the perpendicular plane), two Poisson’s ratios (corresponding to the lateral strains in response to the stresses along the fibers and in the perpendicular plane), and the gel diffusion time (characterizing the fluid motion across the scaffold). The model associates the relaxation mechanism with the fluid diffusion from the surrounding aqueous solution through the porous material, occurring in response to the tensile loading of the cylindrical scaffold. The geometry and structure of the scaffold allows for an adjustment and use of an analytical method previously developed for short tissue (bone, cartilage) cylinders under compression^22^. The quality of the model is subjected to an additional test where the parameters estimated from the experiment corresponding to one strain rate are used for the prediction of the relaxation process corresponding to a different rate and the predicted modeling results are compared with the experiment with the same rate. Finally, the porosity dependence of the scaffold material parameters is estimated. The developed approach can help in the optimization of the experimental and technological efforts associated with the effective development of the scaffolds. After the estimation of the scaffold material parameters, the local stresses, strains, and velocities sensed by stem cells can be obtained as functions of time for different porosities and strain rates, resulting in a more accurate prediction of stem cell differentiation.

## Results

### Biphasic Constitutive Model for the Scaffold Material

In order to reflect the main features of the fibrous-porous structure of the scaffold, we use a biphasic (poroelastic) model of the material. The model assumes that the material is a mixture of two phases, solid and fluid, whose volume fractions are determined by the scaffold porosity. Also, it is assumed that both phases are incompressible, and the solid phase associated with unidirectional fibers is transversely isotropic with isotropic properties in the *x*_*1*_*x*_*2*_-plane normal to the fiber direction (Fig. 1). The main constitutive relations of the model (tensors are shown in bold) take the form^23^

1 $${{\boldsymbol{\sigma }}}^{s}=(1-\varnothing )p{\boldsymbol{I}}+{{\boldsymbol{\sigma }}}^{E}$$

2 $${{\boldsymbol{\sigma }}}^{f}=\varnothing p{\boldsymbol{I}}$$

3 $${{\boldsymbol{\sigma }}}^{{\boldsymbol{t}}}={{\boldsymbol{\sigma }}}^{s}+{{\boldsymbol{\sigma }}}^{f}$$

where **σ**^*s*^, **σ**^*f*^, **σ**^*t*^ and **σ**^*E*^ are the solid, fluid, total, and elastic stress, respectively, ***I*** is the identity tensor, ∅ and *p* are the porosity (fluid volume fraction) and pressure, respectively. The continuity equation for the mixture of incompressible solid and fluid phases takes the form

4 $$div\,[(1-\varnothing ){\bar{v}}^{s}+\varnothing {\bar{v}}^{f}]=0$$

where ${\bar{v}}^{s}$ $\mathrm{and}\,{\bar{v}}^{f}$ are the velocities of the solid and fluid phases (the bars represent vectors), respectively. The solid phase velocity is expressed in terms of the solid phase displacement, ${\bar{u}}^{s}\,,\,\,$as ${\bar{v}}^{s}=\partial {\bar{u}}^{s}/\partial t.$ Neglecting the inertia, the equilibrium equations for the solid and fluid phases, and for the total stress take the form

5 $$-(1-\varnothing )\nabla p+div\,{{\boldsymbol{\sigma }}}^{E}+{\varnothing }^{2}/k({\bar{v}}^{f}-{\bar{v}}^{s})=\,0$$

6 $$-(1-\varnothing )\nabla p-{\varnothing }^{2}/k({\bar{v}}^{f}-{\bar{v}}^{s})=\,0$$

7 $$div\,{{\boldsymbol{\sigma }}}^{t}=0\,$$

where *k* is the permeability of the material.

We treat the scaffold as a transversely isotropic poroelastic cylinder under the action of an axial load resulting in axisymmetric stresses, strains, and velocities. In the cylindrical system (r, $\phi ,z)$) with the (r, $\phi$) coordinates within *x*_*1*_*x*_*2*_-plane, the stresses have σ_*rr*_, σ_*φφ*_ and σ_*zz*_ components, the displacements have ${u}_{r}\,{\rm{and}}\,{u}_{z}$ components, and the strains have ${{\epsilon }}_{rr}=\partial {u}_{r}/\partial r,{{\epsilon }}_{\phi \phi }={u}_{r}/r,{\rm{and}}\,{{\epsilon }}_{zz}=\partial {u}_{z}/\partial z$ components. The scaffold fibers are long with the actual length-to-radius ratios of about 10. Because of this, we assume that ${{\epsilon }}_{zz}=\varepsilon (t)$ (where $\varepsilon (t)$ is the externally applied tensile strain) and other components of the strains, stresses, and displacements do not depend on the z-variable and are functions of the radius, *r* and time, *t*. For infinitesimal strains, the elastic stresses are related to strains by the equations

8 $$\begin{array}{llll}(\begin{array}{c}{\sigma }_{rr}^{E}\\ {\sigma }_{\phi \phi }^{E}\\ {\sigma }_{zz}^{E}\end{array}) & = & [\begin{array}{lll}{C}_{11} & {C}_{12} & {C}_{13}\\ {C}_{12} & {C}_{11} & {C}_{13}\\ {C}_{13} & {C}_{13} & {C}_{33}\end{array}] & (\begin{array}{c}{\varepsilon }_{rr}\\ {\varepsilon }_{\phi \phi }\\ {\varepsilon }_{zz}\end{array})\end{array}$$

The stiffness parameters, *C*_*ij*_ are related to Young’s moduli and Poisson’s ratios by the equations

9 $${C}_{11}={E}_{1}(1-{\upsilon }_{31}^{2}{E}_{1}/{E}_{3})/[(1+{\nu }_{21}){\Delta }_{1}]\,{C}_{12}={E}_{1}({\nu }_{21}-{\upsilon }_{31}^{2}{E}_{1}/{E}_{3})/[(1+{\nu }_{21}){\Delta }_{1}]$$

10 $${C}_{13}={E}_{1}{\nu }_{31}/{\Delta }_{1}\,{C}_{33}={E}_{3}\,[(1+{\upsilon }_{31}^{2}{E}_{1}/{E}_{3})/{\Delta }_{1}]$$

where ${\Delta }_{1}=1-{\upsilon }_{21}-2{\upsilon }_{31}^{2}{E}_{1}/{E}_{3}$. Here, *E*_*3*_ and *E*_*1*_ are Young’s moduli along the fiber direction (in tension) and in the plane of isotropy (in compression), respectively. Also, *υ*_*21*_ and *υ*_*31*_ are Poisson’s ratios given by the following ratios of the strain components ${\upsilon }_{21}=-{\varepsilon }_{\phi \phi }/{\varepsilon }_{rr}$ and ${\upsilon }_{31}=-{\varepsilon }_{rr}/{\varepsilon }_{zz}$. Below, we will analyze the scaffold stress relaxation by considering the time course of the change in the intensity of the tensile load applied to the cylinder (Fig. 1) which is given by the equation

11 $$P(t)=\frac{2}{{a}^{2}}{\int }_{0}^{a}{\sigma }_{zz}^{t}rdr$$

where *a* is the radius of the cylindrical scaffold.

### Optimization of the Model Parameters by Fitting the Bioreactor Relaxation Experiment

The problem under consideration for a long cylinder under tension can be solved by the same method as that previously suggested for a short cylinder under compression^20,22^ resulting in an analytical expression for the load intensity *P(t)* in response to the ramp strain in the form

$$\varepsilon (t)=[\begin{array}{c}{\dot{\varepsilon }}_{0}t\,for\,\,0\le t\le {t}_{0}\\ {\varepsilon }_{0}={t}_{0}{\dot{\varepsilon }}_{0}\quad for\,t > {t}_{0}\end{array}]$$

Below we use this expression (Supplementary Information) to analyze the scaffold relaxation process.

We estimate the five model parameters as follows. As it is shown in the previous section, the elastic parameters of the scaffold are two Young’s moduli, *E*_*1*_ and *E*_*2*_, and two Poisson’s ratios, ${\upsilon }_{21}\,{\rm{and}}\,{\upsilon }_{31}$. The fluid diffusion through the scaffold is characterized by the permeability coefficient, *k*. It is convenient to use the gel diffusion time, *t*_*g*_, instead, as an additional independent parameter because it is directly visible in the experiment. The *t*_*g*_-time is related to the permeability, *k*, by the following equation

$${t}_{g}=\frac{{a}^{2}}{k{C}_{11}}$$

We then are able to reduce the number of parameters involved in the fitting the experimental data. Indeed, the modulus *E*_3_ is the stiffness of the scaffold in the fiber direction under the equilibrium conditions ($t\to \infty )$ i.e.

$${E}_{3}=\frac{{P}_{eq}}{{\varepsilon }_{0}}=\frac{P(\infty )}{{\varepsilon }_{0}}.$$

Also, the bioreactor is accompanied by FDIC software that generates 2-D plots of the equilibrium displacements^3^ . We use these data^3^ to extract Poisson’s ratio ${\upsilon }_{31}$ from the following equation

$${\upsilon }_{31}=-\frac{{\varepsilon }_{rr}}{{\varepsilon }_{zz}}=\frac{\frac{\partial {u}_{r}}{\partial r}}{{\varepsilon }_{0}}$$

which results ${\upsilon }_{31}\approx 0.24$, an estimate that will be used below.

**Figure 2:**
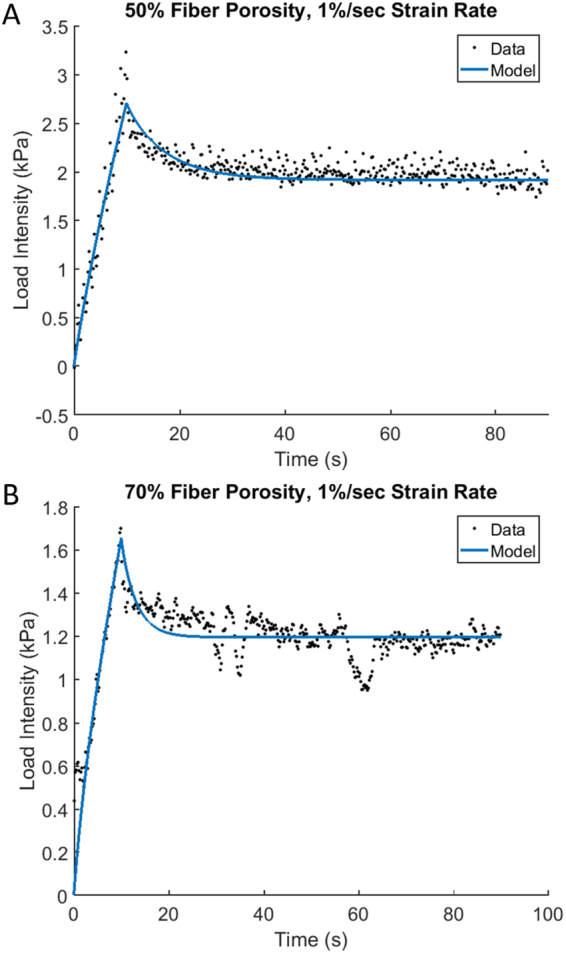
Optimization of the model parameters. The computed time course of the force relaxation (solid line) vs. the experimental data (dots). The total strain, strain rate, porosity are 10%, 1%/s, 50% (**A**) and 10%, 1%/s, 70% (**B**).

Thus, the optimization process is used to extract the material parameters *E*_1_, *v*_21_ and *t*_*g*_ (the computational method of optimization is discussed in the Methods section). The results of the parameter estimation by fitting the experimental data for *ε*_0_ = 10%, *ε*_0_ = 1% s^−1^ and two values of the porosity, 50% and 70% are collected in Table 1. The independently estimated parameters, *E*_*3*_ and ${\upsilon }_{31}$, are included in Table 1 for the completeness. The results show that the scaffold is stiffer in tension along the fiber direction than it is in compression along the radial direction (modulus *E*_*3*_ is about twice larger than modulus *E*_*1*_). Also, the higher porosity results in a softer scaffold in all directions and in shorter gel diffusion time (*t*_*g*_ -time is about twice longer for the smaller porosity of 50% than that in the case of 70%). Figure 2 shows the predicted time-course based on the optimal parameters vs. that measured in the experiment where both have the same strain, strain rate and porosity. The solid lines show the mathematical solution, and the dots represent the experimental measurements (see the Methods section on the treatment of the raw data). Figure 2A and B correspond to two values of the porosity, 50% and 70% respectively. Here and below, the closeness of the modeling results to the experiment are characterized by the r^2^-value which is 0.86 and 0.76 in the case of Fig. 2A and B, respectively.

**Table 1:** The optimal model parameters for the porosities of 50% and 70%.

Porosity	50%	70%
Model Parameters		
E_1_, kPa	8.49	5.61
t_g_, s	40.62	17.58
ν_21_	0.75	0.82
E_3_, kPa	19.19	11.97
ν_31_	0.24	0.24

### Additional Testing of the Model

**Figure 3:**
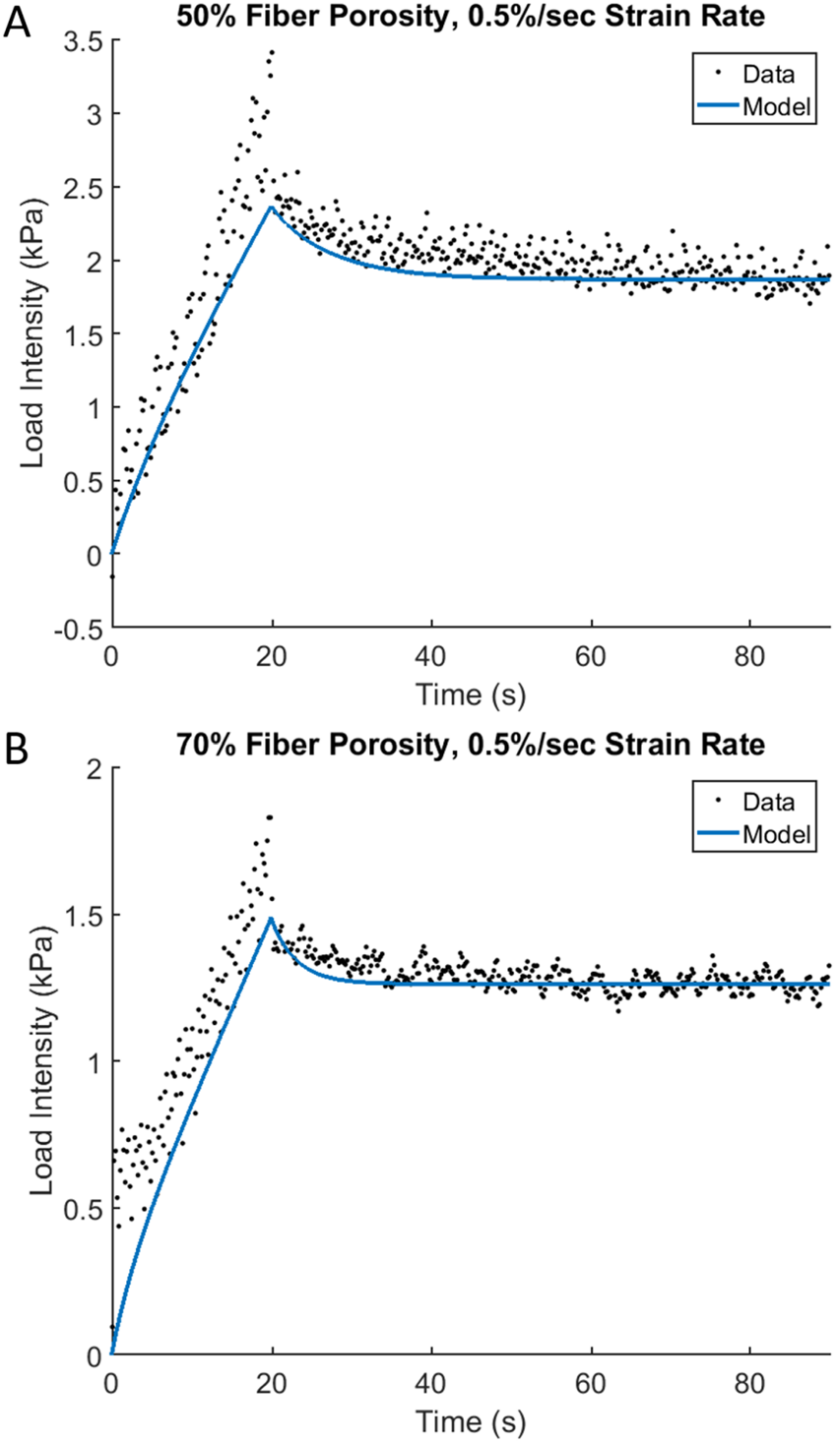
Further validation of the model. The time course (solid line) computed for the optimal parameters vs. experimental data for a different strain rate of 0.5%/s.

After the estimation (optimization) of the model parameters by fitting the experimental data for the strain rate of 1% s^−1^ and two porosities of 50% and 70%, we do an additional test to the model. Since the material parameters of the scaffold do not depend on the strain rate, we consider a different rate of 0.5% s^−1^ and compare the modeling results with the previously optimized parameters against the independent experimental data. The results of this comparison for two porosities of 50% and 70% are presented in Fig. 3A and B, respectively. The solid lines represent the modeling results and the dots correspond to the experimental data (the raw experimental data are treated the same way as in the above case of the strain rate of 1% s^−1^). The value of the total strain is the same as that in Fig. 2 and equal to 10%. The closeness between the modeling and experimental data is characterized by the high r^2^-values which are equal to 0.82 and 0.83 (Fig. 3A,B). In the case of the porosity of 50%, the quality of the model approximation is similar in both loading and relaxation (Fig. 3A). In the second case of the 70%-porosity, the quality of the model approximation during loading is lower although overall r^2^-values are similar.

### Model Predictions of Scaffold Relaxation for Different Strain Rates and Porosities

In the previous sections, we estimated the model parameters by fitting the experimental data corresponding to the strain rate of 1% s^−1^. We additionally tested the parameters by computing the results for another strain rate of 0.5% s^−1^ and comparing them with the experimental data corresponding to that rate. Such estimates were done for two porosities of 50% and 70%. We now use the estimated parameters for predicting the relaxation process as a function of the strain rates.

**Figure 4:**
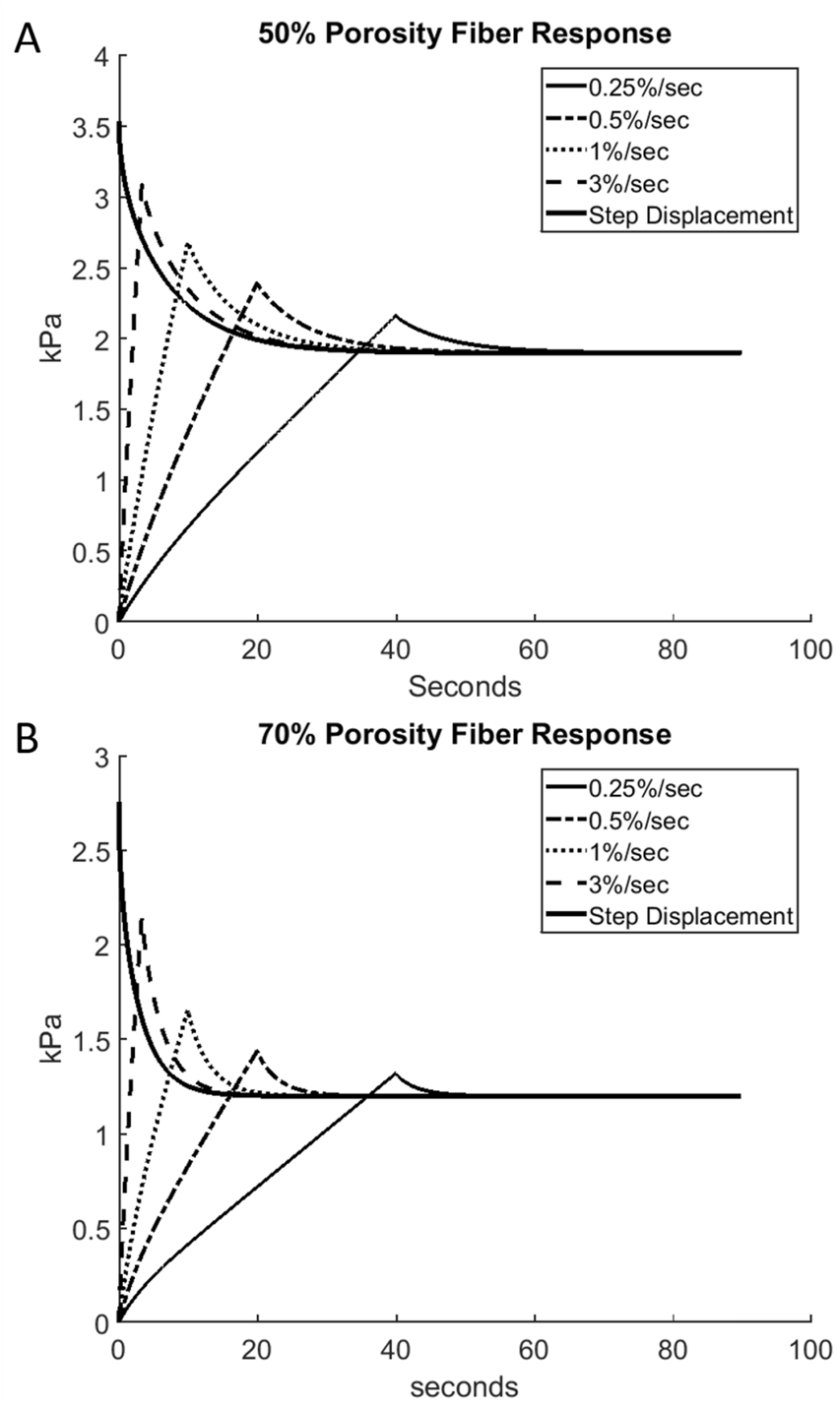
The computed time course of the relaxing force for different strain rates (solid, dashed-dotted, and dashed lines correspond to 0.25%/s, 0.5%/s, 1%/s, and 3%/s, respectively; thick solid line corresponds displacement applied step-wise). (**A**) 50% porosity and (**B**) 70% porosity.

Figure 4 shows the time course of the scaffold relaxation for different strain rates of loading. Figure 4A and B correspond to the porosities of 50% and 70%, respectively. The thin solid, dashed-dotted, dotted, and dashed lines correspond to the loading strain rates of 0.25, 0.5, 1, and 3% s^−1^, respectively. In addition, the thick solid line shows the relaxation curve for the “infinitely large” strain rate which is equivalent to the limiting case of the strain applied step-wise. The modeling parameters are the same (optimal) throughout Fig. 4. The strain rate affects the peak load intensity because the duration of the loading parts is different: it is inversely proportional to the slope of the loading part which provides the same level of the total strain. Thus, the ratios of the peak value of the load intensity to its equilibrium value are equal to 1.86, 1.62, 1.41, 1.26, and 1.14 in the cases of infinite, 3, 1, 0.5, and 0.25% s^−1^ strain rates, respectively, for the porosity of 50%, and they are equal to 2.30, 1.79, 1.38, 1.20, and 1.10 for the same set of strain rates for the porosity of 70%. Although the gel diffusion time, *t*_*g*_, is the same (optimal) for each strain rates, the equilibrium value is reached faster for smaller strain rates because the corresponding peak values are lower.

**Figure 5:**
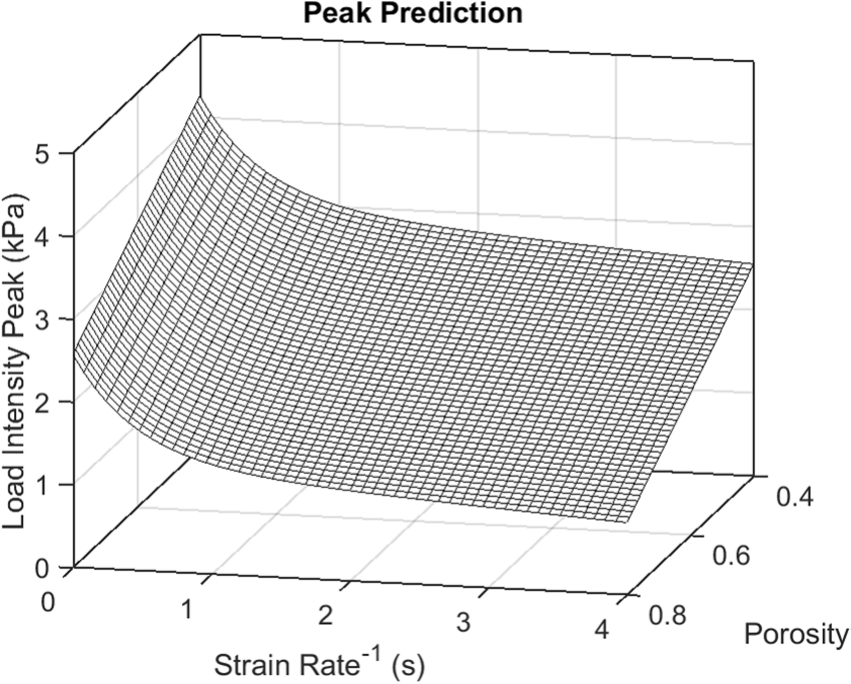
The computed values of the peak force as a function of the porosity and strain rate.

**Figure 6:**
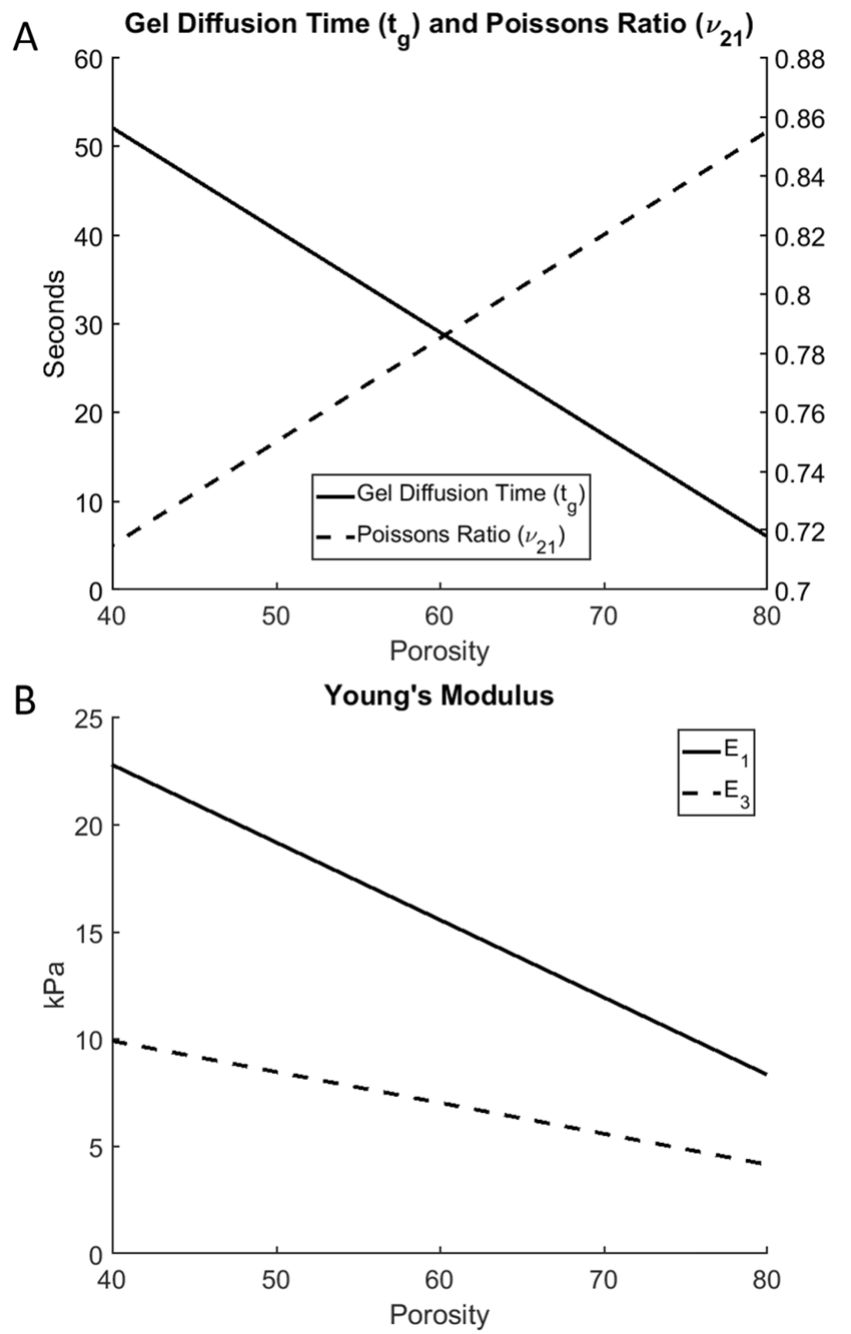
The porosity dependence of the model optimal parameters. (**A**) The gel diffusion time, *t*_*g*_, and Poisson’s ratio, *ν*_*21*_, and (**B**) Young’s moduli, *E*_*1*_ and *E*_*3*_.

We also use the model to predict the relaxation process as a function of the scaffold porosity. For that, we linearly interpolate the model parameters between the previously estimated values for two porosities of 50% and 70%. A unifying 2-D picture of the load intensity peak as a function of the strain rate and porosity is presented in Fig. 5. The function was approximated as

12 $${P}^{pk}=f(x,\,y)=mx+n+[a\cdot exp(by)+c\cdot exp(dy)]$$

where *P*^*pk*^ is the value of the peak and the arguments x and y represent the porosity and strain rate. The values of a = 12.9, b = −2.1, c = −112.2, d = 0.001, m = −4.2, and n = 116.9 are found by fitting the modeling results for several combinations of the porosity and strain rate. Thus, the peak value linearly decreases with porosity and exponentially decreases with the strain rate where its absolute maximal value of about 4 kPa corresponds to the step-wise strain application to the scaffold of minimal porosity of 40%. Finally, Fig. 6 shows the porosity dependence of the scaffold material parameters. Figure 6A shows the porosity dependence of the gel diffusion time (dashed line) and Poisson’s ratio ν_21_. Both of them increase with porosity. Figure 6B presents the porosity dependence of two Young’s moduli, *E*_*1*_ (solid line) and *E*_*3*_ (dashed line), and both of them decrease with porosity.

## Discussion and Conclusions

In this paper, we apply an approach that has been broadly used in the analyses of various properties of tissues. The axial loading of cylindrical specimens allows the effective use of coordinated experimental and modeling methods. Importantly, the mathematical models result in analytical solutions which makes the interpretation of experiments and parameter optimization more transparent than if the solution would have been numerical. The developed analysis takes into account the material anisotropy, biphasic (fluid and solid) structure and the level of the porosity of the scaffold. We have shown here that the relaxation properties of such scaffolds can be explained by the fluid diffusion after the loading of the sample, and we have extended the analyses of short cylinders under compression to those of long cylinders under tension. The mathematical technique used here can be further extended to cover the viscoelastic properties of the solid component (some cases have previously been considered in ref.^25^) and creep regimes (some of them have been considered in refs^24,25^). Also, the technique used allows for computing the strains, stresses, and fluid velocity fields inside the 3D scaffold. Often, in the analysis of the effects of the mechanical factors on stem cell differentiation, the externally applied strain is assumed to be locally sensed by stem cells inside the scaffold. Thus, the computation of the local distributions of the mechanical characteristics gives the knowledge of the true mechanical factors affecting stem cells within localized regions of the scaffold. These local distributions can enter local criteria of stem cell differentiation, like one proposed in ref.^26^ for porous tissues in the form of a linear combination of the velocity of the fluid component and shear strain of the solid component. The scaffold relaxation time has been shown to affect differentiation of the seeded stem cells^15^. Here, the relaxation time is characterized by the model parameter, *t*_*g*_, which depends on the porosity two-fold, via the material permeability and through the ratio of Young’s moduli, *E*_*1*_*/E*_*3*_. However, the actual relaxation to time is not equal to t_g_. First, in contrast to the Maxwell viscoelastic model (e.g.^15^), the poroelastic scaffold has an infinite number of relaxation times represented by the exponential terms in the equation for the load intensity (Supplemental Information). Each of these times is proportional to *t*_*g*_, but is also modified by a factor ${\propto }_{n}^{2}$ (Supplementary Information) weakly dependent on the porosity and strongly increasing with *n*, such that only a few first times are significant to the relaxation. Also, the strain rate has an effect on the actual time of relaxation from the peak value to the equilibrium value of the load which is shown by the set of different relaxation curves corresponding to different strain rates but all of them having the same *t*_*g*_-value.

One of the results of the present paper is the prediction of the effects of porosity and strain rate on the process of scaffold relaxation obtained by using a limited number of computations for a few particular conditions. It can reduce the experimental and technological efforts involved in the study of 3D scaffolds. Finally, we provide additional accuracy of our model by both optimizing the model parameters against experimental data and double-checking the modeling results with previously optimized parameters against the experimental data corresponding to different conditions (strain rate).

In conclusion, the mechanism of relaxation of the fibrous-porous (electrospun fibrin) scaffold is explained using a poroelastic model where the internal fluid diffuses through the solid fibrous matrix in response to the loading until the reaching of the equilibrium state. The material parameters are estimated by fitting the time course of loading-relaxation of the specimen. The relaxation time, important factor of differentiation of the seeded stem cells, is predicted as a function of the scaffold porosity and loading strain rate. The obtained results can help in a more accurate prediction of the effects of the local mechanical factors on stem cell differentiation and in a reduction of the experimental work involved in the study of the properties of 3D scaffolds for regenerative medicine.

## Methods

### Optimization of the Model Parameters

In order to optimize the model parameters, the data need to be processed into a usable form and then curve fitted with the model. To process the raw data to model output, the sensor signal is converted from voltage to force to kilopascals. Due to the low sensitivity of the sensor and the external motor interference, the resulting signal-to-noise ratio is low. The noise needs to be reduced so that it will not distort the model fitting process. One of the most common methods to improve the signal quality without a large loss in the accuracy is to apply a smoothening filter over the experimental data set.

Since the noise originates from two main sources, the cyclic motion of the motor and the noise from the sensor, there is sinusoidal and normal random variations in the data. Analysis of the data demonstrates that the sinusoidal variation is as the largest factor. The periodic noise can be dramatically reduced by the application of a moving average type or equivalent filter. One such filter is the Savitzky-Golay filter^27^. This filter is a linear least squares algorithm that fits subsets of the data with polynomials. The filter is chosen for two reasons. First, a moving average type filter is the equivalent of the filter with a polynomial of degree 1. Second, a prebuilt function already exists in MATLAB as sgolayfilt (). Note that because the ramping section is linear in nature, so a polynomial of degree of 1 is the most suitable for the smoothening process. Exponential relaxation can also be approximated by a linear polynomial for small sections since there is no change in the sign of the slope.

The Savitzky-Golay filter removes any periodic feature in respect to the length of the subset of data involved because, by having an unweighted linear regression on every data point in the subset, the filter should make the smoothened point reflective of the median line of the subset. The frame length can be calculated using the frequency of the sinusoidal noise and the frequency of data collection (100 data points per second) according to the following equation

13 $$Frame\,Length=\frac{Ramping\,Time\,({t}_{0})\,\ast \,Data\,Points\,(\frac{100}{sec})}{Total\,\#\,Cycles\,During\,Ramping\,Period}$$

Once the filter is run on the dataset to smoothen the values, only some minor cyclic noise and random noise will remain. The minor cyclic noise is due to the fact that the MATLAB filter can only implement integral values for the frame length. If the length value comes out to be a decimal, the subset will not be perfect in capturing the periodic noise of the data.

After the processing and smoothening the data, the actual optimization of the model parameters can be done by curve fitting the nonlinear piecewise poroelastic functions to the experimental data. The model involved 5 main parameters, *ν*_*21*_, *ν*_*31*_, *E*_*1*_, *E*_*3*_, and *t*_*g*_. Of these, *ν*_*31*_ and *E*_*3*_ can be calculated from the data and previous studies as discussed previously. The value of *ν*_*31*_, 0.24, is assumed to have little variation and kept constant for each case. E_3_ represents the steady state relaxation value of each case, which can be approximated by taking the average of the last 1000 data points and dividing by the total strain (10% for this study).

To optimize the final 3 parameters, *ν*_*21*_, *E*_*1*_, and *t*_*g*_, the optimization toolbox in MATLAB is used. Since the model is nonlinear and piecewise in nature, the lsqcurvefit () MATLAB function is used for the optimization process. The lsqcurvefit function focuses on non-linear models and uses a least squares method with a default trust-region-reflective algorithm. The least squares method tries to minimize the sum of squared differences (SSD) between the theoretical results and the actual data. The trust region algorithm is involved the exact method of how to find multiple parameters simultaneously. The standard algorithm takes a smaller region N of the target function f, approximates it to a quadratic model q, then calculates steps by minimizing over N. The process is repeated until convergence. The starting point is determined by the user for the algorithm to begin at.

In order to streamline the optimization process, the starting point was visually approximated using plot from predicted theoretical values. This allowed for large steps in the parameter values. Upper and lower bounds were also determined for all the parameters so that the optimization process would not find possible SSD minimums that were outside possible values. For example, the bounds for t_g_ and E_1_ are relative to the exponential slope of the relaxation section and the maximum intensity value respectively which can be roughly estimated. The tolerances involved in finding the minimum and the parameters steps were also increased by a factor of two degrees from the MATLAB default. The increase decreased the optimization dramatically (by a similar factor as the increase) with minimal loss in accuracy.

After optimization of the first data set, steps were taken to further decrease computational time. Since ν_21_ should be somewhat consistent over all the different porosities, the value could be held initially from the optimization so that the process only involves 2 parameters, which is much less computationally intense. The resulting two values should be much closer to the actual optimized values than the original starting point. These two values for *E*_*1*_ and *t*_*g*_ could then be fed back into the optimization with ν_21_ to recalculate values for the data set in less steps. Additionally, since *t*_*g*_ is related to relaxation time, after the first optimization of *t*_*g*_, the latter *t*_*g*_ values could be initially chosen slightly easier since the relationship was better understood.

There was also some optimization of the characteristic function calculation. The characteristic function involved calculating the zeroes of a function that involved Bessel functions. MATLAB has a function that can emulate the Bessel function, besselj (). This was used in conjunction with fzero() to calculate the zeroes. However, there are an infinite number of zeroes possible starting from 0. Every zero is required to compute the exact value of the characteristic function. After some analysis of the results, we chose 20 iterations as the suitable number of zeroes for the characteristic function calculation because there was zero variation in the final value compared to 21 iterations at this point.

### Scaffold Relaxation Experiment

All stress relaxation experiments were performed in a custom-built bioreactor^2,3^. The bioreactor allows for testing in an aqueous environment, thus allowing for the observation of the poroelastic stress relaxation of the fibrin specimens. The specimens of various porosities were clamped between a fixed force sensor (Cooper Instruments 50 gram max load cell) and a mobile linear actuator controlled by a linear stepper motor (Haydon Kerk). The clamping was achieved through custom 3D printed clamps. Force measurements were taken through the force sensors which are attached to a strain gauge amplifier unit (Industrologic SGAU). The voltage output from this system was read through a Lab Jack U3-HV and was recorded in real time through Lab Jack’s custom software. The specimens were loaded at their resting length and then an additional 1 mm of slack length was provided to ensure that there was no pre-tension in the fiber. Following this, the end of the fiber attached to actuator was retracted 10% of the original length. During this period, the real-time force was recorded to establish the 0% position of the fiber, so that the fiber was not slack or pre-tensioned before the relaxation experiment began. Following the pre-tensioning, fibers were extended to 10% of their adjusted length and held at 10% strain for 100 seconds to allow for measurement of stress relaxation through the force sensor readout. The 10% strain was applied at various strain rates, controlled through the stepper motor.

### Availability of Materials and Data Statement

The results are made transparent and reproducible by the derivation of the model, including Supplementary Information. The estimation of the model parameters is supported by the detailed description (including the names of software subroutines) in the Methods section. The experimental methodology is described in the same section. There are no specific datasets used in the paper.

## Electronic supplementary material

Supplementary Information
