# Supplementary material for: A Poroelastic Model of a Fibrous-Porous Tissue Engineering Scaffold
Source: Sci Rep. 2018 Mar 22;8:5043. doi: 10.1038/s41598-018-23214-8 (PMC5864912; doi:10.1038/s41598-018-23214-8)
Supplement: Supplementary file 1 — Supplementary Information [file 41598_2018_23214_MOESM1_ESM.pdf]

A Poroelastic Model of a Fibrous-Porous Tissue Engineering Scaffold

Daniel Yuan, Sarah M. Somers, Warren L. Grayson & Alexander A. Spector

Supplementary Information

Supplementary Information

The main steps of the derivation of the load intensity, *P(t),* are as follows. First, the continuity equation (4) is used to obtain the fluid velocity, in terms of the radial displacement, u. Then, the obtained fluid velocity is used in the equilibrium equation (6) to derive pressure, p, in terms of the radial displacement, u. Thus, the problem reduces to a second-order ODE in terms of u-displacement as a function of r and t. Using Laplace transform over the time, t, the problem reduces to a Bessel equation in terms of the Laplace transform of u. The inverse Laplace transform is obtained by calculating the residues. Finally, the longitudinal stress, ,is obtained in terms of the displacement, u, and integrated over the scaffold cross-section resulting in the following expression for the load intensity

$$P(t) = E_3\dot{\varepsilon_0}\,t + E_1\dot{\varepsilon_0}\,t_g F_1\big(t_g, E_1, E_3, \upsilon_{21}, \nu_{31}\big) \text{ for } 0 \leq t \leq t_0 \quad \text{(S1)}$$

$$P(t) = E_3\dot{\varepsilon_0}\,t_g - E_1\,\dot{\varepsilon_0}\,t_g F_2\big(t_g, E_1, E_3, \upsilon_{21}, \nu_{31}\big) \text{ for } t \geq t_0 \quad \text{(S2)}$$

Where:

$$F_1 = \Delta_3\left\{\frac{1}{8} - \sum_n \exp\left(-\alpha_n^2 \frac{t}{t_g}\right)\left(\propto_n^2 \left[\Delta_2^2 \propto_n^2 - \Delta_1\ (1+\nu_{21})\right]\right)^{-1}\right\}, \quad \text{(S3)}$$

$$F_2 = \Delta_3 \sum_n \left[\exp\left(-\alpha_n^2 \frac{t}{t_g}\right) - \exp\left(-\alpha_n^2 \frac{t-t_0}{t_g}\right)\right]\left(\propto_n^2 \left[\Delta_2^2 \propto_n^2 - \Delta_1\ (1+\nu_{21})\right]\right)^{-1} \quad \text{(S4)}$$

$$\Delta_2 = \left(1 - \nu_{31}^2 \frac{E_1}{E_3}\right) \text{ and } \Delta_3 = \left(1 - \frac{2\nu_{31}^2 \Delta_2}{\Delta_1}\right) \quad \text{(S5)}$$
